# Efficient shoot regeneration of medicinal plant *Haplophyllum tuberculatum* by direct and indirect organogenesis and genetic fidelity assessment using Inter Simple Sequence Repeats markers

**DOI:** 10.3389/fpls.2022.995825

**Published:** 2022-09-21

**Authors:** Mohammed Alsafran, Kokila Wickramanayake, Kamal Usman, Talaat Ahmed

**Affiliations:** ^1^Agricultural Research Station, Office of VP for Research and Graduate Studies, Qatar University, Doha, Qatar; ^2^Central Laboratories Unit, Office of VP for Research and Graduate Studies, Qatar University, Doha, Qatar; ^3^Office of Academic Research, Qatar University, Doha, Qatar; ^4^Environmental Science Center, Qatar University, Doha, Qatar

**Keywords:** *Haplophyllum tuberculatum*, shoot regeneration, ISSR marker analysis, medicinal plant, PGR

## Abstract

*In vitro* plant cell and tissue cultures are potent tools to propagating germplasm resources in conserving and managing plant genetic resources. A reliable micropropagation protocol was developed for efficient callus proliferation and direct and indirect shoot regeneration of Meseika (*Haplophyllum tuberculatum*). With the applied sterilization procedure, immature, unopened *H. tuberculatum* seed pods can be identified as a potent explant with high viability and low contamination percentage. Multiple shoots were regenerated from leaf and stem explants through direct organogenesis on Murashige and Skoog’s (MS) + 3% sucrose medium amended with BAP. Indirect regeneration of several shoots was achieved on 1/2 MS + 1% sucrose media amended with 2 and 4 mg/l BAP. An efficient callus proliferation from both explants can be achieved by supplementing the MS media with NAA and BAP. All the cultures were incubated in a controlled growth chamber under 5/19 h light/dark photoperiod, temperature (25 ± 2°C), and 60% relative humidity (RH).10 ISSR (Inter Simple Sequence Repeat) markers were screened to test the genetic fidelity of regenerated *H. tuberculatum* shoots. Callus development was observed after 15 days and shoot regeneration was occurred after 30 days after callus initiation. 10 ISSR primers produced a total of 39 clear, distinct amplicons. 75, 60, 40, and 16% polymorphism percentages were recorded by the ISSR primer 11, 7, 5, and 4, respectively. The developed micropropagation protocol is appropriate for rapid *in-vitro* multiplication of *H. tuberculatum* shoots and callus.

## Introduction

In desert ecosystems where water is considered the primary limiting factor, some physiological traits such as seed germination of drought-resistant xeric plants are critically influenced by seasonal hydric conditions during the winter months. However, there was a significant reduction even in the scarce rainfall that occurs during the winter season of Qatar. This has a significant detrimental effect on Qatar’s dessert floral diversity, one of the reservoirs of fascinating adaptations. It is pivotal to develop and implement efficient germplasm conservation strategies such as micropropagation to save such vital gene pools.

**Figure 1:**
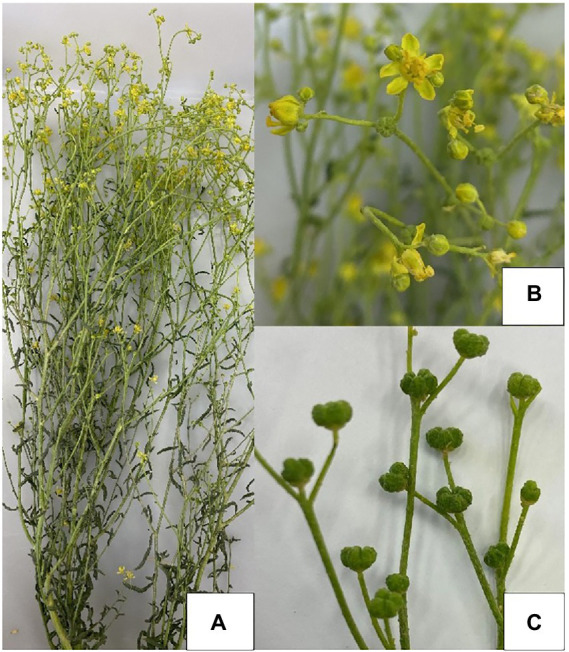
Meseika (*H. tuberculatum*), Doha, Qatar. **(A)** Whole plant, **(B)** Flowers, **(C)** Immature seed pods.

*Haplophyllum tuberculatum* L. belongs to the family Rutaceae and is a perennial shrub with habitats in most Mediterranean countries and the Arab peninsula (Raissi et al., 2016; Flora of Qatar, 2021). *H. tuberculatum* is a flowering shrub that grows about 40–60 cm with green stems. The leaves are alternate and elliptic to obviate (Al-Muniri and Hossain, 2017). The flowers are arranged in loose terminal panicles, and mature seed pods bear dark brown to brownish-black tinny seeds (Figure 1). This shrub is armed with a characteristic pungent odor to repel herbivores.

Oman, Sudan, and Saudi Arabia, *H. tuberculatum* plays a significant role in folk medicine (Raissi et al., 2016). *H. tuberculatum* is used to treat the nervous system, infertility, gynecological disorders, fever, and remedy headaches and arthritis (Raissi et al., 2016). Plant leaves and stems contain different classes of secondary metabolites such as alkaloids, furanocoumarins, lignans, and flavonoids (Al-Muniri and Hossain, 2017). Accessions in Oman contain essential oils rich in b-phellandrene, limonene, b-ocimene, a-caryophyllene, myrcene and a-phellandrene (Eissa et al., 2014; Al-Saeghi et al., 2022).

Antioxidant and antimicrobial assays done with the different plant extracts and essential oil derived from the areal parts of *H. tuberculatum* showed promising results as a potential source of antioxidants and antimicrobial compounds (Al-Burtamani et al., 2005; Eissa et al., 2014; Al-Muniri and Hossain, 2017; Al-Saeghi et al., 2022). These have been shown to possess radical scavenging activities with a potential to alleviate diseases neurodegenerative disorders induced by reactive oxygen species (Al-Burtamani et al., 2005). Extracts have also demonstrated antifungal and antiviral properties against *Fusarium culmorum, Rhizoctonia solani*, and tobacco mosaic virus (TMV), having a high concentration in resveratrol kaempferol, myricetin, rutin, quercetin, and rosmarinic acid (Chandran et al., 2020). Thus organ cultures or cell cultures will be a potential source to produce and extract bioactive compounds on an industrial scale (Chandran et al., 2020; Wawrosch and Zotchev, 2021).

In general, wild medicinal plant resources have enormous pressure on their natural habitats and threaten to become endangered due to several factors. Global warming and reducing annual rainfall is a major determinant of plant growth reproduction and has significantly impacted seed production, the percentage of seed germination, and seed viability. At the same time, overgrazing has dramatically reduced seed production. Further aspects such as its use as firewood and the increase in urbanization have compounded the decline of *H. tuberculatum* populations.

Therefore, the knowledge derived on *H. tuberculatum* micropropagation is timely and pivotal for resilient conservation and germplasm management, improving the wild accessions, and producing plant materials for plant materials extraction of phytochemicals in the future as required. *In-vitro* regeneration of *H. tuberculatum* has not received adequate attention in the literature. Only few studies evaluated the micropropagation potential of *H. tuberculatum* using axillary buds, shoot tips, and single-node cuttings (Almazrooei, 2018; Abdallah et al., 2019).

Although plants are considered totipotent, there is a potential risk of generating spontaneous somaclonal mutants when maintaining *in vitro* cultures (The National Academies Press, 1993). In this regard, DNA marker analysis was done to check the genetic fidelity of the regenerated plantlets using Inter Simple Sequence Repeats (ISSR) markers. ISSR marker analysis can be identified as a relatively easy, reliable, cost-effective, and efficient technique to assess the genetic fidelity of the micro propagated plants, which has been extensively used in the literature (Alansi et al., 2016; Thorat et al., 2018). As this method is independent of DNA sequence data availability, many scientists have used the technique with various underutilized or wild plant species (Alansi et al., 2016).

Nevertheless, this is the first attempt at direct and indirect regeneration of *H. tuberculatum*, starting with leaf explant and assessment of the genetic fidelity of the regenerated *H. tuberculatum* plantlets. Apart from that, particularly in Qatar, no attempt has been made to micropropagate *H. tuberculatum* accession available in Qatar to date. This study presents the findings of callus induction and shoot regeneration of *Hapllophyllum tuberculatum* (Meseika) Qatar accession through direct and indirect organogenesis and the assessment of genetic fidelity of the regenerated shoots using Simple Sequence Repeats (ISSR) Markers.

## Materials and methods

### Plant materials

The Ministry of Municipality and Environment supplied us with a freshly harvested, well-grown *H. tuberculatum* plant sample (Figure 1), which was used to excise the explants for the initial establishment of *in vitro* cultures. The plant species (*H. tuberculatum*) was identified accurately using guidelines from the text book “An Illustrated Checklist of the Flora of Qatar” (Norton et al., 2009) and verified by a plant taxonomist. All the explants for the rest of the experiments were obtained from the *in vitro* raised plantlets (Armijos-González et al., 2021).

### Media and culture conditions

MS (Murashige and Skoog, 1962) medium with or without PGRs (Plant Growth Regulators) containing sucrose as a carbon source was used as the basal media. The pH of the culture medium was adjusted to 5.7 ± 0.1 with NaOH or HCl before gelling with 3 g/l Gellan Gum. The medium was autoclaved at 121°C for 20 min. All the cultures were incubated in a controlled growth chamber under 5/19 h light/dark photoperiod, temperature (25 ± 2°C), and 60% relative humidity (RH).

### Surface sterilization and evaluation of the effect of 2,4-D (2,4-Dichlorophenoxyacetic acid) and BAP (6-Benzylaminopurine) on *Haplophyllum tuberculatum* immature seeds

Surface sterilization was done according to the method described by Abdallah et al. (2019) with some modifications. In short, unopened immature seed pods were immediately collected into a beaker filled with tap water to maintain the freshness of the explants. Before transferring to the laminar flow chamber, explants were gently washed and rinsed with running tap water for 30 min to eliminate surface residues, followed by a quick rinse with a commercial liquid soap. Then the explants were treated with 25% Clorox® containing 2 drops of TWEEN® 20 for 25 min, followed by a quick dip in 70% ethanol for 20 s. Traces of the commercial bleach and ethanol was entirely removed by rinsing the explants with sterilized distilled water after each treatment.

Surface sterilized unopen immature *H. tuberculatum* seed pods were aseptically divided into a single lobe used as the initial explant. MS + 3% sucrose as the basal media with three concentrations (i.e., 0.5, 1, 2 mg/l) of 2,4-D and BAP was used in the callus induction experiment. Each treatment was replicated ten times. The number of contaminated and survived explants were recorded after 5 days. And the degree of callus formation was scored based on the extent of growth of the callus biomass: light (1), moderate (2), moderate profuse (3), profuse (4) growth as recorded after 15 days.

### Indirect regeneration

This stage aimed to increase the number of proliferated shoots. The callus biomass was recovered from already established callus cultures. An experiment was conducted with different PGRs at different concentrations to select the best media for shoot generation. 1 g of callus biomass was initiated on a 1/2MS basal medium containing 1% sucrose, 0.3% Gellan gum, and pH 5.7 ± 0.1 and supplemented with different concentrations of IBA (Indole-3-butyric acid), IAA (Indole-3-acetic acid), Kinetin, and BAP. Each treatment was replicated 10 times. Fresh weight increase, color and nature of callus, and degree of shoot regeneration were recorded after 45 days. After 30 days from callus initiation, the shoots with leaves only were considered when determining the visible shoot-out growth; the green-colored calluses were excluded.

### Effect of different combinations of auxins and cytokinin on leaf and stem explants

MS basal medium containing 3% sucrose, 0.3% Gellan gum, and pH 5.7 ± 0.1 supplemented with two auxins (2,4-D and NAA (1-Naphthaleneacetic acid)) and cytokinin (BAP) individually and in combination to make 36 different media was evaluated for callus growth and organogenesis. *In vitro* regenerated leaves and stems were used as explants. The observations were recorded after 45 days (Table 1).

**Table 1:** Combinations of auxins and cytokinin used in initiation media with leaf and stem explants.

		NAA (mg/L)			
		0	1	2	4
	0	Ctrl	C1	C2	C3
2,4-D (mg/L)	1	C4	C5	C6	C7
	2	C8	C9	C10	C11
	4	C12	C13	C14	C15
		**BAP (mg/L)**			
		0.5	1	2	
	0	C16	C17	C18	
	1	C19	C20	C21	
	2	C22	C23	C24	
	4	C25	C26	C27	
	1	C28	C29	C30	
NAA (mg/L)	2	C31	C32	C33	
	4	C34	C35	C36	

Ctrl; free hormone media, C1–C36 are the combination between each two hormones. For examples; C1 means 1 mg/l NAA + 0 mg 2,4-D, while C36 means 2 mg/l BAP + 4 mg/l NAA.

### ISSR maker analysis

Genomic DNA extraction was done using regenerated single shoots. And a verified *H. tuberculatum* plant from the desert was used as the reference plant (positive control). About 2 cm regenerated 14 shoots finely ground using mortar and pestle. And leaves from the reference plant were finely ground using mortar and pestle with liquid N2. Genomic DNA was extracted using the QIAGEN DNeasy PowerSoil® DNA extraction kit. The concentration of the gDNA samples was measured by NanoPhotometer® N60. The PCR with each ISSR primer was prepared as described below.

The PCR reactions were carried out in a 25 μl volume containing 15 ng genomic DNA, 13.5 μM primer (synthesized from IDT), 12.5 μl Thermo Scientific Dream Taq PCR Master Mix (2x). The reactions were set up in ProFlex PCR system. The PCR program consisted of initial denaturation at 95°C for 1.5 min, followed by 40 cycles of 30 s denaturation at 95°C, 30 s annealing at the optimized annealing temperature for each primer (Table 2), and 1 min extension at 72°C. This was followed by a 7 min final extension at 72°C. ISSR PCR amplificants were electrophoresed through 1.5% Agarose gel for 35 min at 100 V. The bands were recorded into the binary symbols, 1 for band presence, whereas 0 for band absence. Only the clearly distinctive bands were scored.

**Table 2:** List of different ISSR (Inter Simple Sequence Repeat) primers used for detecting clonal stability of regenerated *H. tuberculatum*, melting and annealing temperatures, scoring results of the amplicon profiles.

Primer code	Primer No.	*T*_m_ (°C)	Annealing temp. (°C)	Primer sequence (5′-3′)	Number of scorable bands	Polymorphic bands	Polymorphism %	Approximate rage of amplicon size (bp)
60,154,438	3	47.6	42	(CA)6 AC	1	0	0	800–1,000
60,154,439	4	46.7	42	(CA)6 GT	6	1	16	200–3,000
60,154,440	5	43.6	42	(CA)6 AG	5	2	40	400–3,000
60,154,442	7	41.9	40	(GA)6 GG	5	3	60	400–3,000
60,154,445	10	44.7	42	(CAC)3 GC	2	0	0	400–1,500
60,176,223	11	50.5	46	(CT)8 GC	4	3	75	700–1,500
60,176,225	13	41.0	40	(GAG)3 GC	5	0	0	200–3,000
60,176,227	15	44.0	42	(GTG)3 GC	7	0	0	300–3,000
60,176,228	16	54.2	46	(AGA GAG)3 AGC	2	0	0	1,000–2000
60,176,229	17	52.4	46	(CTC TCT)3 CTG	2	0	0	800–1,500
					Total 39	9		Average 200–3,000

### Statistical analysis

All the above experiments were laid in the completely randomized design (CRD) in a controlled growth chamber. The data was analyzed, and the graphs were generated by IBM SPSS Statistics 28.0.0.0 software. The Quantitative data were analyzed with analysis of variance (ANOVA) followed by mean comparison using Tukey HSD at 0.05 confidence level.

## Results

### Surface sterilization and evaluation of the effect of 2,4-D and BAP on *Haplophyllum tuberculatum* immature seeds

Unopened immature *H. tuberculatum* seed pods were selected as the explant to avoid damaging tissues of tinny seeds by direct exposure to the disinfectants. The surface sterilization method resulted in a high viability percentage of 94–100% and a lower contamination percentage of 1%. The explants on the media containing 2,4-D and BAP at all three different concentrations (i.e., 0.5, 1, and 2 mg/l) initiated green-colored callus at a different growth rate (Table 3). Moderate to profuse callus growth was recorded on the media contained 2,4-D where the explants initiated on media with BAP there was light callus growth recorded.

**Table 3:** Effectiveness of the surface sterilization method and *H. tuberculatum* immature seed explant response to 2,4-D and BAP different concentrations.

PGR and concentration	Viability %	Degree of callus growth	Callus color
0.5 mg/l 2,4-D	100	Moderate profuse	Green
1 mg/l 2,4-D	100	Profuse	Green
2 mg/l 2,4-D	100	Profuse	Green
0.5 mg/l BAP	100	Light	Green
1 mg/l BAP	94	Light	Green
2 mg/l BAP	100	Light	Green
Overall contamination	1.0%		

2,4-D (2,4-Dichlorophenoxyacetic acid), BAP (6-Benzylaminopurine).

### Indirect regeneration

**Figure 2:**
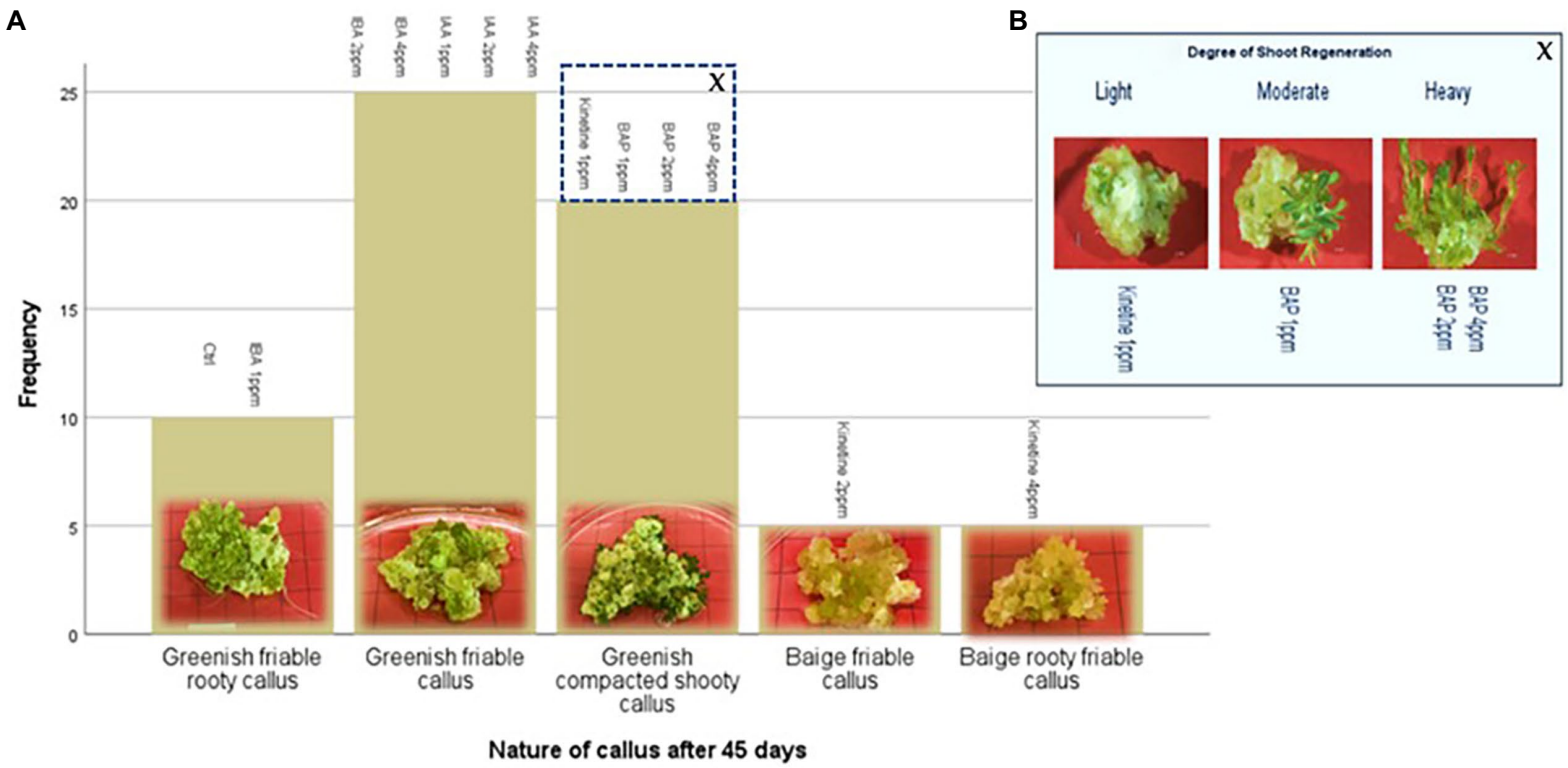
Indirect regeneration of shoots; **(A)** Nature of callus after 45 days on regeneration media, **(B)** Degree of shoot regeneration. Each treatment was replicated 10 times.

**Figure 3:**
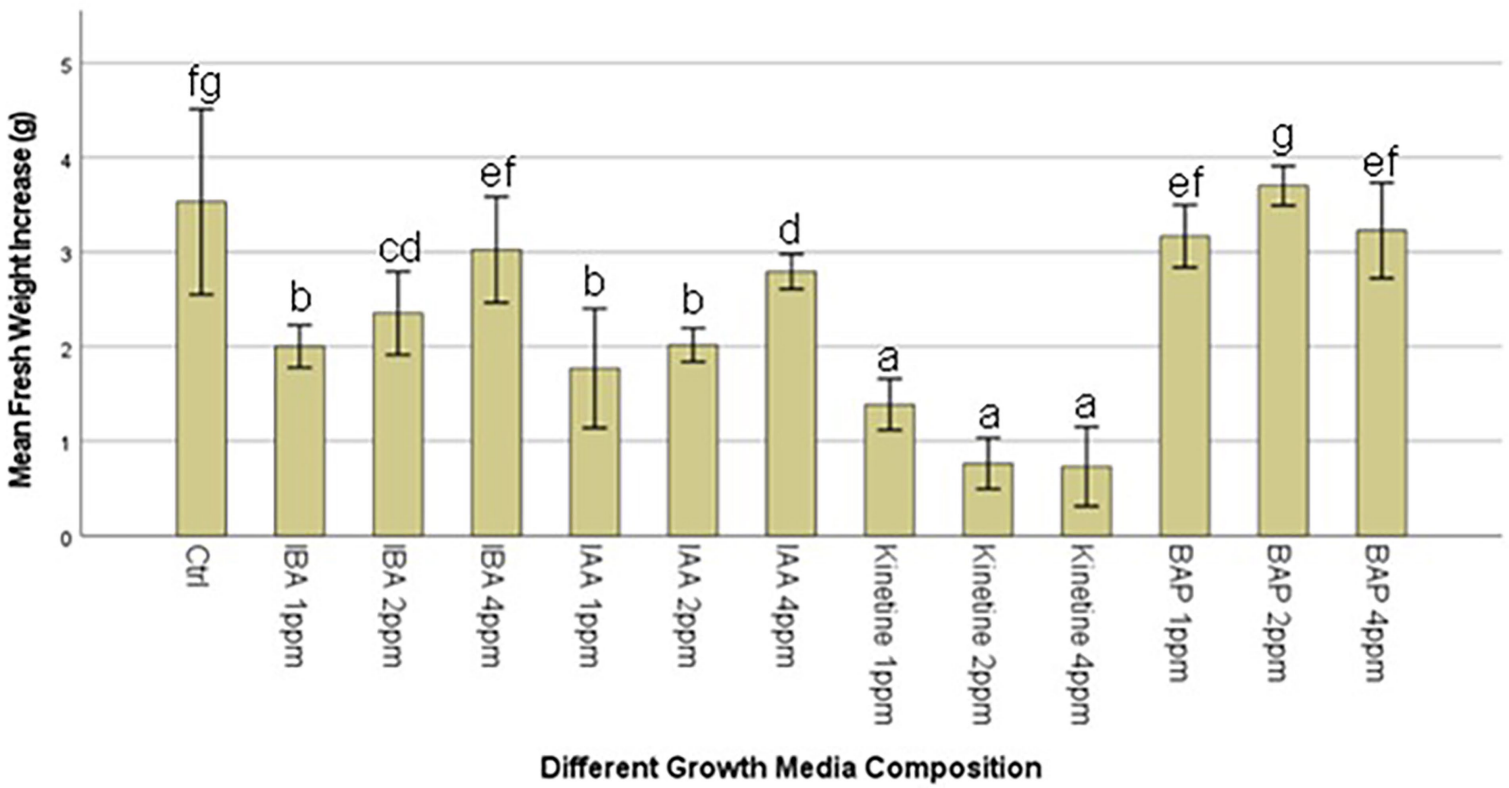
Average callus fresh weight increase in 1/2MS + 1% sucrose with varying IBA (Indole-3-butyric acid), IAA (Indole-3-acetic acid), Kinetine, and BAP (6-Benzylaminopurine) levels.

This stage aimed to increase the number of proliferated shoots from the callus, and the results are presented in Figures 2A,B. An experiment was conducted with two different auxins and cytokines, namely IBA, IAA, Kinetin, and BAP, respectively, at three concentrations of 1,2 and 4 mg/l to evaluate the effect on callus multiplication rate, regeneration, and morphology. 1/2MS containing 1% sucrose, 0.3% Gellan gum, and pH 5.7 ± 0.1 was used as the basal media in this experiment. The control had only the basal media without any PGRs. 1 g of callus, which was maintained in 2 mg/l 2,4-D, was initiated on each media. The highest fresh weight increase was recorded in 2 PPM BAP-containing media. However, this was not significantly different from the control. Statistically, the lowest fresh weight increase was recorded at all concentrations of Kinetin followed by the next lowest was recorded with IAA. There was no statistically significant difference between the different concentrations of IAA. Though IBA recorded a higher fresh weight increase than Kinetine and IAA, it was not higher than the fresh weight increase recorded in control. However, 4 ppm IBA resulted in a statistically similar fresh weight increase as in control and BAP except with 2 ppm BAP which recorded the highest fresh weight increase (Figure 3).

The callus underwent different morphological changes in different media. The callus initiated on media containing kinetin 2 ppm and 4 ppm remained a beige friable callus. Among them, 4 ppm Kinetine resulted in rooty callus (Figure 2A). Greenish callus resulted in all other media, including the control. Among them, callus started shoot re-generation had become compact. BAP induced shoot regeneration at all three concentrations and Kinetine at 1 ppm (Figure 2A). The shooty callus was rated according to the degree of shoot regeneration as light, moderate and heavy (Figure 2B). Heavy shoot regeneration was recorded at high BAP concentrations, 2 ppm and 4 ppm, than at 1 ppm BAP. T.

### Effect of different combinations of auxins and cytokinin on leaf and stem explants

**Figure 4:**
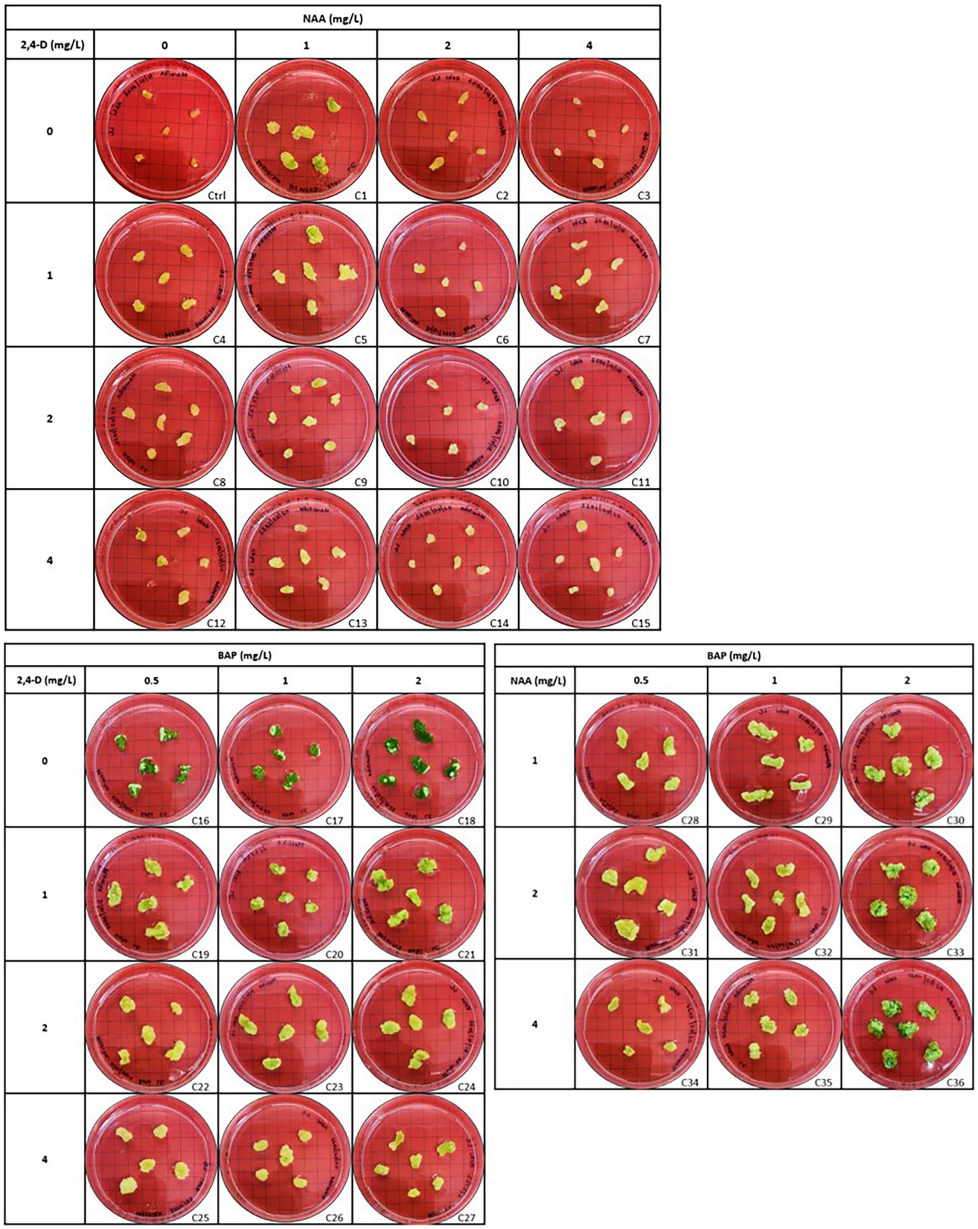
Morphogenic response of leaf explants in different concentrations and combinations of 2,4-D (2,4-Dichlorophenoxyacetic acid), NAA (1-Naphthaleneacetic acid) and BAP (6-Benzylaminopurine) 45 days after initiation. Each treatment was replicated 10 times.

**Figure 5:**
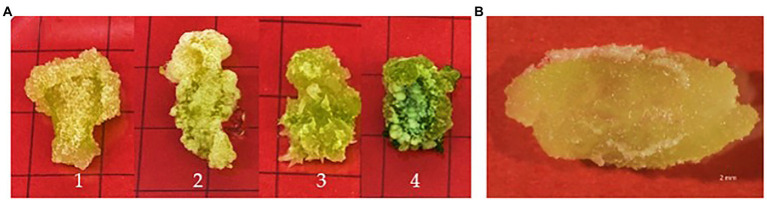
**(A)** Different textured *H. tuberculatum* callus in response to different combinations of PGRs (Plant Growth Regulators): (1) Friable callus, (2) Compact callus, (3) Rooty callus, (4) Compacted green callus, **(B)** Callus induction from leaf explant as whole plant development into a proliferating cell mass. Each treatment was replicated 10 times.

**Figure 6:**
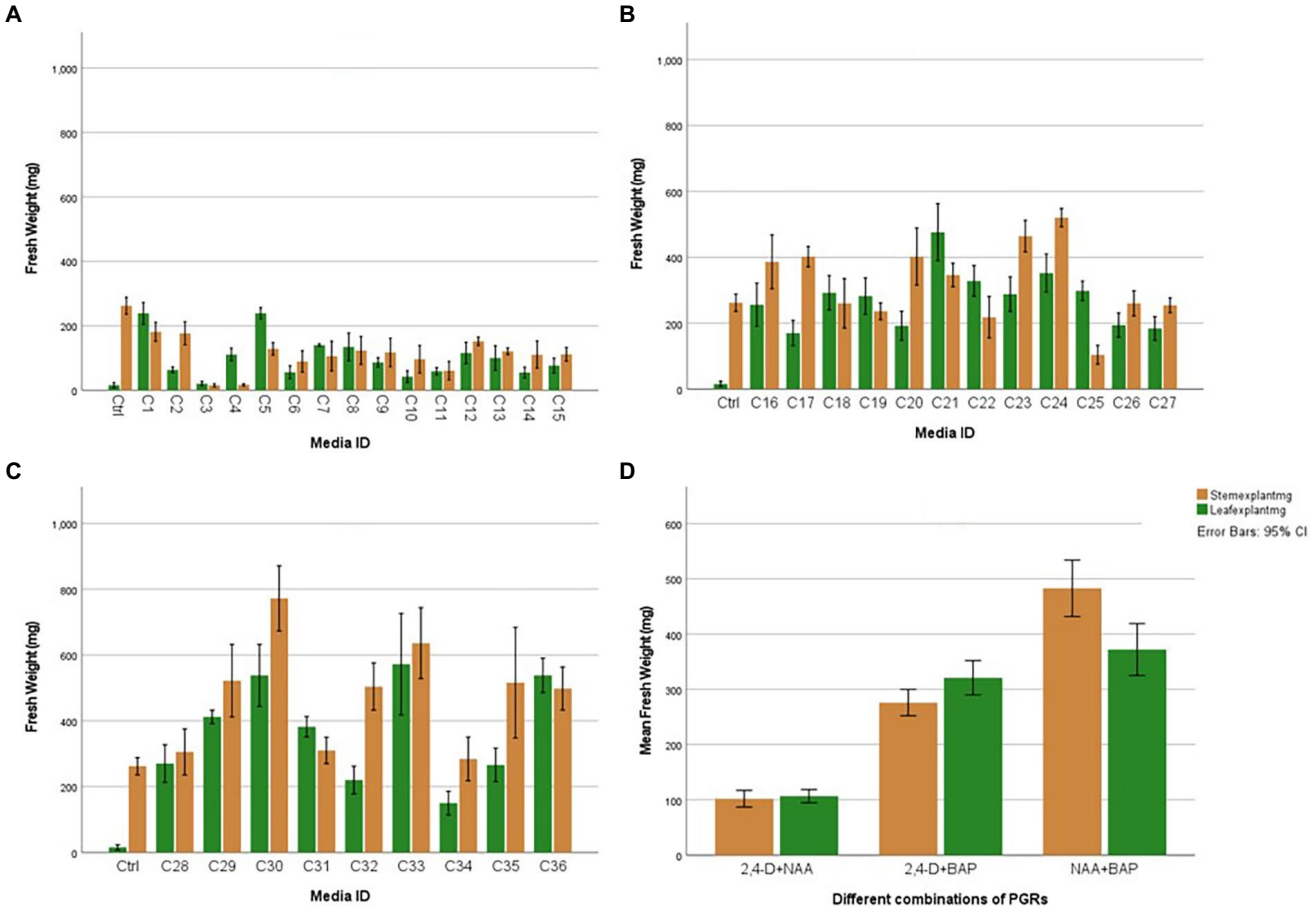
Average fresh weight of leaf and stem explants after 45 days with individual and different combinations of 2,4-D (2,4-Dichlorophenoxyacetic acid), NAA (1-Naphthaleneacetic acid), and BAP (6-Benzylaminopurine): **(A)** Effect of 2,4-D (2,4-Dichlorophenoxyacetic acid) and NAA (1-Naphthaleneacetic acid), **(B)** Effect of 2,4-D (2,4-Dichlorophenoxyacetic acid) and BAP (6-Benzylaminopurine), **(C)** Effect of NAA (1-Naphthaleneacetic acid) and BAP (6-Benzylaminopurine), **(D)** Overall Explant response. Each treatment was replicated 10 times.

Callus initiation efficiency response of leaf and stem explants was studied by initiating them on the media supplemented with 2,4-D, NAA, and BAP at different concentrations independently and in combination. The results are presented in Figures 4–6. Leaves and stems excised from *in vitro* regenerated *H. tuberculatum* shoots were used as the explants in this experiment. There was a wide range of variations in callus texture, color, degree of callus formation, and regeneration (Figure 4). Leaf explants initiated on basal media without PGRs did not produce callus or organs. However, the stem ex-plants produced auxiliary shoots in the same control media. All the combinations of Auxin and Cytokinin were designed to get high auxin concentration with low Cytokinin concentration except in media C21 and C30 to induce callus proliferation (Figure 4).

The nature of callus development was observed as whole explant conversion into a proliferating cell mass regardless of callus initiation from a cutting edge or a midrib (Figure 5B). The ANOVA resulted in a statistically significant difference with both stem and leaf explant and the Tukey HSD test was conducted to compare multiple means separately for each explant type. Even though the highest fresh weight increases with the leaf explant in the media amended with NAA + BAP, it is not statistically different from the fresh weight increase with 2,4-D + BAP. In particular, stem explant resulted in the highest fresh weight of 772 mg in the media containing 2 mg/l BAP + 1 mg/l NAA can be suggested as the best media for efficient *H. tuberculatum* callus proliferation. Overall fresh weight increase was higher in the media with Auxin and Cytokinin. However, the highest fresh weights of 772 mg and 636 mg were recorded in the media C30 and C33, containing 2 mg/l BAP + 1 mg/l NAA and 2 mg/l BAP + 2 mg/l NAA, respectively, with stem explants. The leaf explants recorded the highest fresh weight of 572 mg and 538 mg in the media C33, C36 and C30 containing 2 mg/l BAP + 2 mg/l NAA, 2 mg/l BAP + 1 mg/l NAA and 2 mg/l BAP + 4 mg/l NAA, respectively.

### ISSR (Inter Simple Sequence Repeat) maker analysis

**Figure 7:**
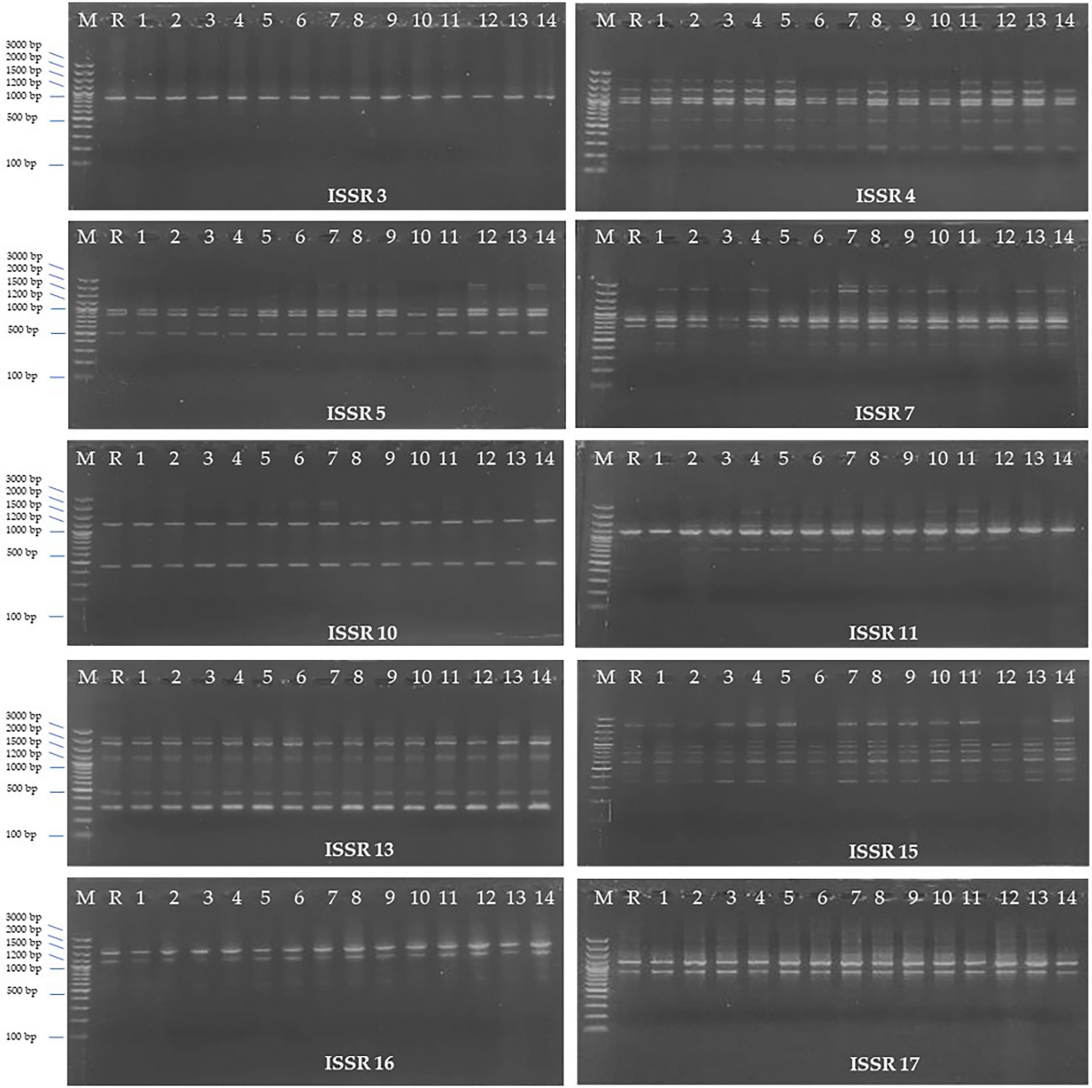
ISSR (Inter Simple Sequence Repeat) profiles of 10 ISSR primers; Lane 1: Ladder, Lane 2: Reference plant, Lane 3–14: regenerated *H. tuberculatum* shoots.

ISSR marker analysis was carried out to evaluate the genetic fidelity of the regenerated *H. tuberculatum* shoots. ISSR profile of the 14 regenerated shoots from indirect organogenesis and the reference plant (Figure 7R) with 10 ISSR primers has been shown in Figure 7. Ten primers produced a total of 39 fragments ranging from 1 (ISSR 3) to 6 fragments (ISSR 4) with an average frequency of 3.9 bands per primer (Table 2). The size of amplicons with the 10 ISSR primers varied from 200 to 3,000 bp. The results showed that 30 bands were monomorphic, and only 9 amplified bands were polymorphic. 75, 60, 40, and 16% polymorphism percentages were recorded by the ISSR 11, 7, 5, and 4, respectively.

## Discussion

In conservation and bulk production of plant materials for bioactive compounds, the development and implementation of micropropagation methods can play a vital role because the conventional methods are time-consuming and often exposed to biotic and abiotic stresses. *H. tuberculatum* is a promising source of antioxidants and antimicrobial compounds. However, its biodiversity is threatened by climate change and other human practices. Here we were able to construct an effective protocol for its *in vitro* regeneration and propagation to assist its conservation and potentiate efficient production of its valuable natural chemicals.

The effectiveness of the surface sterilization depends on various factors such as likely contaminations, type of explant, donor plant raised environment, etc. The common issues of surface sterilization are either disinfection treatments not being robust to eliminate bacterial and fungal contaminants or the explant tissues get severely damaged and result in a significantly low explant survival. Several disinfectants, such as Ethanol, Ca(ClO)2, NaOCl, H2O2, AgNO3, HgCl2, Silver nitrate, etc., are commonly used for surface sterilization of explants at the different immerse times (Hesami et al., 2019). In this study, the surface sterilization method was developed based on the method described by Abdallah et al. (2019) but was slightly modified by excluding the treatment of the explant with mercuric chloride (HgCl2) due to its toxicity (Abdallah et al., 2019). Despite not using HgCl2, we were able to achieve a nearly 100% efficacy of disinfection on the explants. The used surface sterilization method resulted in a high viability percentage of 94–100% and a lower contamination percentage of 1%. The resulted in higher viability percentage may be due to use of unopened *H. tuberculatum* seedpods which avoided damaging tissues of tinny seeds by direct exposure to the disinfectants. MS + 3% sucrose as the basal media with both the media amended with 1 and 2 mg/l 2,4-D can be used to initiate *H. tuberculatum* callus with profuse callus growth.

The immature unopened *H. tuberculatum* seedpods were selected as the best explant in this study to initiate cultures from the mother plants obtained from the desert. Similarly, Lucke (1971) successfully sterilized tinny orchid seeds while they were capsulated in an immature capsule without losing their viability (Zhao et al., 2010). Deng et al. (2020) obtained an efficient callus production from immature lotus cotyledon and embryo explants grown on Murashige and Skoog (MS) basal medium containing 3 mg/l 2,4-D and 0.5 mg/l BAP. Zhao et al. (2010) achieved the highest embryogenic callus induction frequencies of 57 and 74% from the seeds of two different sweet sorghum cultivars on MS basal media supplemented with 4 mg/l 2,4-D. By using unopened *H. tuberculatum* seedpods we avoided damaging tissues of tinny seeds by direct exposure to the disinfectants, thus increasing their viability and culture initiation potential. This was evident by the observed explant viability of nearly 100% under the different hormone treatments.

The effect of 2,4-D and BAP individually was evaluated on immature *H. tuberculatum* seed explants. In general, 2,4-D is critical for callus induction (Neibaur et al., 2008). Our results indicated that the degree of callus growth from *H. tuberculatum* immature seeds is moderate profuse to profuse with 2,4-D, while there was only a light callus growth resulting with BAP. This is in agreement with previous research, where optimal callus induction on MS medium was achieved with 2 mg/l 2,4-D (Wawrosch and Zotchev, 2021). Although a high concentration of 2,4-D resulted in profuse callus growth, a high concentration of 2,4-D can decrease regeneration frequency, and calluses tend to remain in the callus proliferation stage. And there is an increased risk of the development of somaclonal variations under elevated levels of 2,4-D in growth media (Armijos-González et al., 2021), which was also observed during our experiments after assessing regenerated plants with ISSR markers. Therefore 2,4-D used for *H. tuberculatum* calli production is probably one of the major factors that affect the genetic fidelity of regenerated shoots. Thus the observed somaclonal variations in regenerated shoots may be due to the heterogeneity of regenerated callus cells and seeds (Armijos-González et al., 2021). In other Haplophyllum species such as Haplophyllum virgatum var. virgatum, optimal callus generation was achieved with a combination of 0.1 mg/l Kinetin and 5 mg/l IAA in a B5 medium (Abdallah et al., 2019). In Haplophyllum gilesii callus generation was achieved in MS media containing 0.25 mg/l kinetin, 2.5 mg/l BAP and 2.5 mg/l NAA (Almazrooei, 2018). Thus, the use of IAA or NAA instead of 2,4-D should further be examined, especially in genetic conservation work, as genetic fidelity is paramount.

Elmaghabi et al. (2017) successfully regenerated *H. tuberculatum* from sterilized single nodal segments through indirect organogenesis. Successful callus proliferation was achieved on MS medium supplemented with 1 or 2 mg/l 2.4-D and with maltose as the carbon source. They further achieved successful shoot generation from callus cultures could be achieved on MS medium supplemented with 1 mg/l Kinetin (Elmaghabi et al., 2017). Abdallah et al. (2019) used *H. tuberculatum* shoot-tip and single node cutting on MS medium supplemented with 1.0 mg/l Kinetin combined with 0.1 mg/l NAA to acquire multiple shoots. Callus on MS medium supplemented with 1 mg/l Kinetin resulted in significantly higher shoot regeneration. A similar result was reported by Elmaghabi et al. (2017). In our study, the callus initiated on the media containing 2 and 4 ppm BAP showed heavy shoot regeneration. Thus it can be used effectively similarly to Kinetin.

Agha et al. (2022) evaluated the effects of BAP, NAA, and 2,4-D on vitro callus formation from fenugreek seeds. They achieved the highest amount of callus in the media containing both BAP and NAA combinations. In Haplophyllum gilesii the highest amount of callus growth was achieved with, 2.5 mg/l BAP and 2.5 mg/l NAA (Almazrooei, 2018). We reached similar results in our study with *H. tuberculatum*, in terms of BAP and NAA combinations, with both leaf and stem explants. Furthermore, our observations showed that stem explants generated significantly higher callus biomass than leaf explants in the same media (Figure 6D).

*In vitro* morphogenetic response of leaf and stem explants were studied using 2,4-D, NAA, and BAP at different concentrations independently and in combination. Stem explants exhibited the same morphogenic response except on basal medium without PGRs (Control; not presented in results). Stem explants generated auxiliary shoots on basal medium. Both explants induced direct regeneration of shoots (Figure 4) when media contained only BAP. Different PGRs influenced the color and texture of the callus. Furthermore, the intensity of shoot regeneration was increased with increasing BAP concentration. There was no effect of BAP hormone individually on induced callus proliferation of *H. tuberculatum* either from leaf or stem explants (Figure 4). With increasing concentrations of BAP to 2 mg/l direct shoot, regeneration was significantly increased (Figure 4) with both leaf and stem explants.

According to Agha et al. (2022) findings with Fenugreek, though increasing concentrations of BAP increased shoot proliferation, the quality of plantlets was negatively affected (Agha et al., 2022). These findings reinforce the general acceptance of its known function in micropropagation as the stimulant of the growth of axillary, adventitious, and foliar buds (Pereira et al., 2018). Although BAP stimulates shoot proliferation in many plants, it can cause high somaclonal variation rates at high concentrations. Thus, it can be linked to the somaclonal variation we observed in our study. Alternatively, to reduce somaclonal variation, the use of BA for shoot regeneration should be evaluated as it resulted in optimal shoot induction and regeneration at a concentration of 2 mg/l, in the closely related species Haplophyllum virgatum var. virgatum (Abdallah et al., 2019).

Antioxidant and antimicrobial assays with essential oils derived from *H. tuberculatum* showed promising results as a potential source of antioxidants and antimicrobial compounds (Al-Burtamani et al., 2005). In this study, *H. tuberculatum* callus and shoot cultures were initiated quickly, schematically presented in Figure 3. With such fast regeneration and propagation times, calluses at regenerating stage and shoot cultures can efficiently extract essential oil and bioactive compounds for their pharmaceutical and antimicrobial properties (The National Academies Press, 1993; Al-Burtamani et al., 2005; Chandran et al., 2020; Gemmill and Grierson, 2021).

The micropropagation technique is often used to generate cell cultures, organ cultures, and whole plants with similar genetic makeup. Although the literature has shown that seed culturing is a promising technique for *in vitro* multiplication of endangered medicinal plant species, unawareness of the level of genetic purity of the seeds is a barrier to implementing the method for *in-vitro* production of the true-to-type cultures (Attanayaka et al., 2021). Additionally, *in-vitro* cultures are exposed to high levels of oxidative stress. This can lead to chromatin structural changes by chromosome breakage and rearrangement, DNA methylation, point mutations, histone modifications, etc., creating a potential risk of producing somaclonal variations (Krishna et al., 2016). Molecular markers have been used as a powerful tool to detect those somaclonal variations. As genetic markers such as ISSR, SCoT, RAPD require no sequence information and are simple, cost-effective, and confirmed to be effective in assessing the genetic fidelity and genetic diversity, makes them popular among scientists (Kudikala et al., 2020).

In the present study, gDNA of 14 randomly selected *in vitro* regenerated shoots and a reference plant was subjected to ISSR maker analyses to evaluate the genetic fidelity. ISSR marker analysis has been used for similar studies to assess the genetic fidelity of *in vitro* regenerated medicinal plant species (Lata et al., 2013; Yadav et al., 2013; Kudikala et al., 2020). More than one marker type is often recommended to analyze better the genetic fidelity of micropropagated plants (Kudikala et al., 2020). However, in species such as *H. tuberculatum* where genomic information is not available, ISSRs are the most robust, stable, and with the highest degree of polymorphisms, thus the most suitable for use in this species. Our findings on somaclonal variation also support the notion that tissue culture is a stressful environment for the plants and can result in genetic changes in the propagated material (Alansi et al., 2016; Thorat et al., 2018). A further evaluation of the use of less differentiated tissues such as the pericycle and further research on fine-tuning 2,4-D treatments, or finding alternative optimal hormone combinations can help further reduce somaclonal variation and increase genetic fidelity.

## Conclusion

We have developed for the first time an efficient *in-vitro* method for multiple shoot regeneration in an *H. tuberculatum* Qatar accession. The previous disinfection protocol of *H. tuberculatum* was successfully modified, eliminating the use of the toxic HgCl2, achieving very high disinfection efficiencies. Using immature seed pods, we achieved the most profuse callus induction on MS + 3% sucrose media containing 2 mg/l 2,4-D. Further optimal callus proliferation was achieved on ½ MS supplemented with 2 mg/l BAP + 2 mg/l NAA. Callus induction with 2,4-D, however resulted in a small percent of explants exhibiting somaclonal variation as assessed with ISSR markers. Thus, to move forward, fine tuning of the callus initiation protocol is needed with regards to 2, 4-D concentration or use of other auxin types and stricter monitoring with molecular markers to detect and exclude somaclonal variants during *in vitro* propagation. Our results provide the means for effective *H. tuberculatum* conservation and bulk production of bioactive compounds through the implementation of micropropagation methods.

## Data availability statement

The original contributions presented in the study are included in the article/supplementary material, further inquiries can be directed to the corresponding author.

## Author Contributions

TA and MA: conceptualization and resources. KW and TA: methodology and experimental design. KW: writing—original draft preparation and formal analysis and investigation. MA, TA, and KU: writing—review and editing and supervision. KU: project administration. MA: funding acquisition. All authors have read and agreed to the published version of the manuscript.

## Funding

This research was made possible by Qatar University’s Qatar-Japan Research Collaboration Grant (M-QJRC-2020-10). The statements made herein are solely the responsibility of the authors.

## Conflict of interest

The authors declare that the research was conducted in the absence of any commercial or financial relationships that could be construed as a potential conflict of interest.

## Publisher’s note

All claims expressed in this article are solely those of the authors and do not necessarily represent those of their affiliated organizations, or those of the publisher, the editors and the reviewers. Any product that may be evaluated in this article, or claim that may be made by its manufacturer, is not guaranteed or endorsed by the publisher.
